# Correction: Regulating red-shifted phosphorescence of carbon dots *via* introducing magnesium chloride

**DOI:** 10.1039/d6sc90074g

**Published:** 2026-04-07

**Authors:** Yibin Long, Haoda Zhang, Yongjin Chen, Xiaoming Yang

**Affiliations:** a College of Pharmaceutical Sciences, Southwest University Chongqing 400715 P. R. China ming4444@swu.edu.cn; b Center for High Pressure Science and Technology Advanced Research (HPSTAR) Beijing 100193 P. R. China yongjin.chen@hpstar.ac.cn

## Abstract

Correction for ‘Regulating red-shifted phosphorescence of carbon dots *via* introducing magnesium chloride’ by Yibin Long *et al.*, *Chem. Sci.*, 2026, https://doi.org/10.1039/d5sc09281g.

The authors regret that an incorrect version of Fig. 5 was included in their published article. Specifically, Fig. 5e was inadvertently duplicated and placed in the panel representing Fig. 5d, which caused Fig. 5d to be obscured by Fig. 5e, resulting in an error in the image.

To address this issue, the authors have provided the corrected version of Fig. 5, containing the correct image for Fig. 5d, which is shown below.

**Fig. 5:**
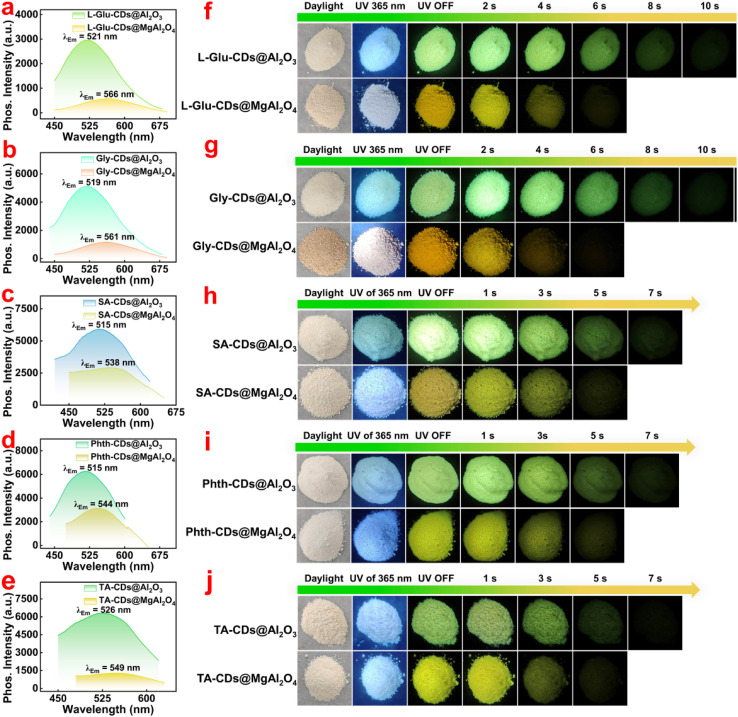
Phosphorescence emission spectra of (a) l-Glu-CDs@Al_2_O_3_ and l-Glu-CDs@MgAl_2_O_4_, (b) Gly-CDs@Al_2_O_3_ and Gly-CDs@MgAl_2_O_4_, (c) SA-CDs@Al_2_O_3_ and SA-CDs@MgAl_2_O_4_, (d) Phth-CDs@Al_2_O_3_ and Phth-CDs@MgAl_2_O_4_, (e) TA-CDs@Al_2_O_3_ and TA-CDs@MgAl_2_O_4_. (f–j) The corresponding images under daylight, 365 nm UV irradiation and after the UV light was turned off.

This correction does not affect the results, interpretation, or conclusions of the study.

The Royal Society of Chemistry apologises for these errors and any consequent inconvenience to authors and readers.

